# Low levels of monkeypox virus-neutralizing antibodies after MVA-BN vaccination in healthy individuals

**DOI:** 10.1038/s41591-022-02090-w

**Published:** 2022-10-18

**Authors:** Luca M. Zaeck, Mart M. Lamers, Babs E. Verstrepen, Theo M. Bestebroer, Martin E. van Royen, Hannelore Götz, Marc C. Shamier, Leanne P. M. van Leeuwen, Katharina S. Schmitz, Kimberley Alblas, Suzanne van Efferen, Susanne Bogers, Sandra Scherbeijn, Guus F. Rimmelzwaan, Eric C. M. van Gorp, Marion P. G. Koopmans, Bart L. Haagmans, Corine H. GeurtsvanKessel, Rory D. de Vries

**Affiliations:** 1 Department of Viroscience, Erasmus University Medical Center, Rotterdam, Netherlands; 2 Department of Pathology, Erasmus University Medical Center, Rotterdam, Netherlands; 3 Department Infectious Disease Control, Municipal Public Health Service Rotterdam–Rijnmond (GGD Rotterdam), Rotterdam, Netherlands; 4 Research Center for Emerging Infections and Zoonoses, University of Veterinary Medicine Hannover, Hannover, Germany

**Keywords:** Viral infection, Live attenuated vaccines, Antibodies

## Abstract

In July 2022, the ongoing monkeypox (MPX) outbreak was declared a public health emergency of international concern. Modified vaccinia Ankara—Bavarian Nordic (MVA-BN, also known as Imvamune, JYNNEOS or Imvanex) is a third-generation smallpox vaccine that is authorized and in use as a vaccine against MPX. To date, there are no data showing MPX virus (MPXV)-neutralizing antibodies in vaccinated individuals nor vaccine efficacy against MPX. Here we show that MPXV-neutralizing antibodies can be detected after MPXV infection and after historic smallpox vaccination. However, a two-shot MVA-BN immunization series in non-primed individuals yields relatively low levels of MPXV-neutralizing antibodies. Dose-sparing of an MVA-based influenza vaccine leads to lower MPXV-neutralizing antibody levels, whereas a third vaccination with the same MVA-based vaccine significantly boosts the antibody response. As the role of MPXV-neutralizing antibodies as a correlate of protection against disease and transmissibility is currently unclear, we conclude that cohort studies following vaccinated individuals are necessary to assess vaccine efficacy in at-risk populations.

## Abstract (web summary)

Historic smallpox vaccination and monkeypox virus (MPXV) infection elicit MPXV-neutralizing antibodies, but MPXV-neutralizing antibodies are less frequent and of lower magnitude after vaccination with MVA-BN—the vaccine approved and in use for protection against MPXV and smallpox.

## Main

MPXV belongs to the *Orthopoxvirus* genus of the Poxviridae family of large double-stranded DNA viruses and causes a zoonotic disease known as MPX. In May 2022, MPX was identified in several countries in which MPX cases had not been reported previously, after which MPXV rapidly spread in Europe and the United States among individuals who had not traveled to endemic areas^1^. On 23 July 2022, this ongoing MPX outbreak was declared a public health emergency of international concern by the Director-General of the World Health Organization^2^.

MPXV is closely related to variola virus, the causative agent of smallpox. Smallpox was eradicated by the use of different attenuated poxvirus vaccines combined with active case finding, isolation and quarantine measures. The first-generation and second-generation smallpox vaccines contained infectious vaccinia virus (VACV) grown in the skin of live animals (for example, Dryvax), the chorioallantoic membrane of eggs (for example, VACV Elstree) or cell culture (for example, ACAM2000)^3^. The third-generation smallpox vaccine was based on an even further attenuated VACV obtained by serial passage in chicken embryo fibroblasts (CEFs), known as modified vaccinia virus Ankara (MVA). Since MVA was developed in the endgame of smallpox eradication (the first market authorization was obtained in Germany in 1977)^4^, its efficacy against smallpox has been inferred based on the non-inferiority of immunogenicity in clinical studies^5^. A study in the Democratic Republic of the Congo suggested that the VACV smallpox vaccine was also effective against MPX to a certain extent^6^. However, efficacy data of the third-generation MVA smallpox vaccine against MPX in humans are lacking. MVA vaccination afforded protection against severe MPX disease and death in non-human primates by inducing both VACV-neutralizing antibodies and T cells, but sterile immunity was not achieved, and some skin lesions remained. The presence of MXPV-neutralizing antibodies in non-human primates was not assessed^7–9^. Partly because of this evidence for protection against severe disease in non-human primates, MVA-BN was licensed as a vaccine against MPX in humans in Canada (known as Imvamune) and the United States (known as JYNNEOS) and was recently approved by the European Medicines Agency under special circumstances (known as Imvanex), despite a lack of efficacy data against human MPXV infection or demonstrable MPXV-neutralizing antibodies in vaccinated individuals.

Randomized trials, test-negative studies and cohort studies are being initiated to better understand the efficacy of MVA-BN against MPX^10^. While these studies are underway, it is equally important to improve understanding of the immunogenicity of MVA, especially with regard to MPXV. Assays to measure VACV-binding and MVA-binding antibodies have previously been developed in enzyme-linked immunosorbent assay (ELISA) formats using purified intracellular mature virions (IMVs) or infected cell lysates^7,11^. Functional antibody measurements through virus neutralization assays using VACV expressing or MVA expressing reporter proteins have been used for studies assessing the non-inferiority of vaccine-induced immunogenicity between new-generation and old-generation vaccines^7,11,12^. However, assays to measure MPXV-specific antibodies are lacking. In the present study, we developed both an ELISA and a neutralization assay based on an MPXV isolate from a Dutch patient and used them in combination with an ELISA with VACV Elstree-infected cell lysate and an MVA-based neutralization assay to address three crucial questions. (1) Are antibodies induced by historic smallpox vaccination cross-reactive with MPXV? (2) Do MPXV-infected individuals rapidly mount neutralizing antibody responses? (3) Does MVA-BN vaccination induce MPXV-reactive and neutralizing antibodies?

## Results

### Historic smallpox vaccination cross-neutralizes MPXV

**Fig. 1:**
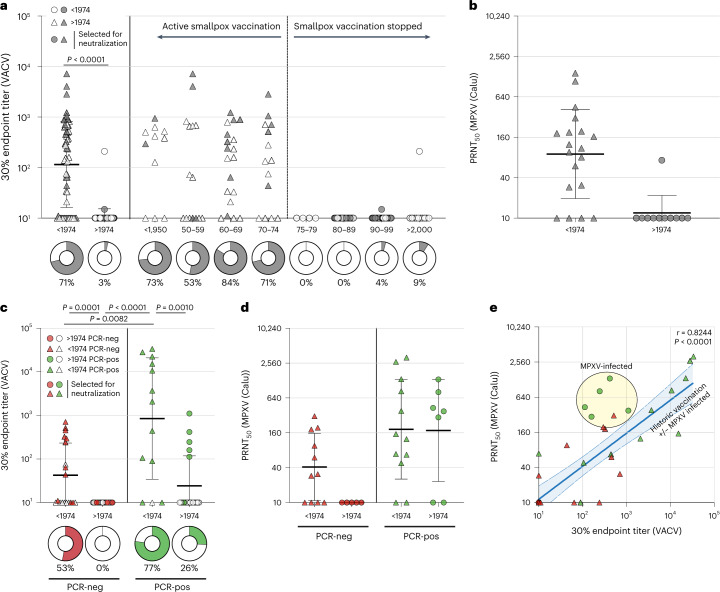
VACV-reactive and MPXV-neutralizing antibodies after historic smallpox vaccination and MPXV infection. **a**,**b**, Detection of poxvirus-specific antibodies in an age-panel of *n* = 126 biologically independent sera. **a**, Detection of VACV-reactive antibodies by ELISA with VACV-Elstree-infected cell lysate. Sera obtained from individuals born in or before 1974 (triangles) or after 1974 (circles) are merged on the left side of the graph and shown per decade on the right side of the graph. Colored symbols reflect sera selected for neutralization assays. Donut graphs show seroconversion percentages. **b**, Detection of MPXV-neutralizing antibodies by PRNT on a selection of *n* = 30 sera. **c**–**e**, Detection of poxvirus-specific antibodies in a diagnostic panel of sera. **c**, Detection of VACV-reactive antibodies by ELISA with VACV-Elstree-infected cell lysate. A total of *n* = 72 sera were obtained from individuals born in or before 1974 (triangles) or after 1974 (circles), who were either PCR negative (red) or PCR positive (green). Colored symbols reflect sera selected for neutralization assays. Donut graphs show seroconversion percentages. **d**, Detection of MPXV-neutralizing antibodies by PRNT on a selection of *n* = 35 sera. **e**, Relationship between VACV-reactive binding and MPXV-neutralizing antibodies by correlating the data from **c** and **d**. 30% endpoint ELISA titers were calculated based on a five-fold dilution series, after subtraction of OD_450_ values against a mock-infected cell lysate and relative to a positive control. The PRNT_50_ titer was calculated on the basis of a two-fold dilution series relative to an infection control. The lines indicate the geometric mean, and the whiskers indicate the 95% confidence interval. Mann–Whitney *U*-tests were performed to compare VACV-reactive endpoint titers (two-tailed *P* < 0.05 was considered significant for **a**, and *P* < 0.0083 was considered significant after Bonferroni correction for multiple comparisons in **c**; comparisons not leading to a significant difference are not shown). VACV-reactive and MPXV-neutralizing antibodies were correlated by performing Spearman’s *r* analysis (excluding the *n* = 5 sera from MPXV-infected individuals born after 1974).

**Extended Data Fig. 1:**
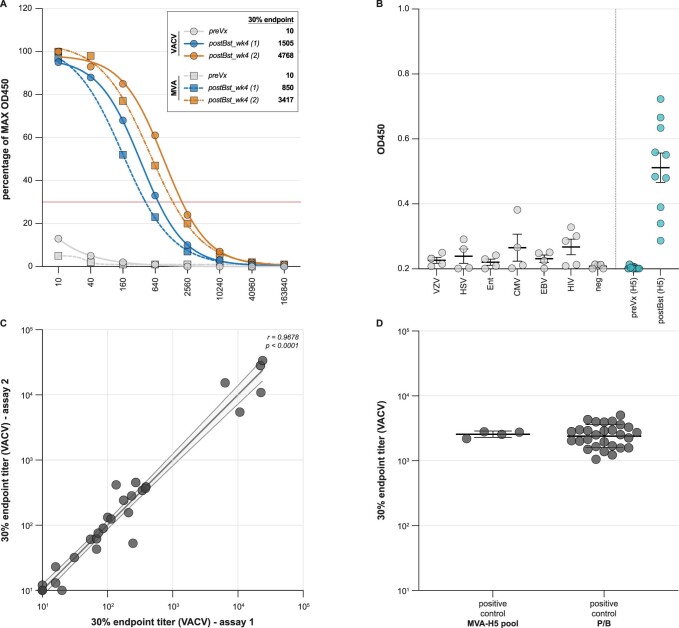
Validation of ELISA with VACV-Elstree-infected cell lysate. (**a**) Sera from participants vaccinated with MVA-H5 (pre-vaccination and 4 weeks after booster vaccination) were 4-fold diluted starting at a dilution of 1:10 and added to plates coated with a VACV-infected or MVA-infected cell lysate. All sample OD450 values were normalized to the highest and lowest OD450 value on that plate and plotted as log (inhibitor) vs response curve with four parameter variable slope using GraphPad Prism 9.02. A 30% endpoint dilution was estimated by calculating the proportionate distance between two dilutions, from which a titer was determined. 30% endpoint titers are reported in the legend for both ELISAs with VACV-infected and MVA-infected cell lysate. (**b**) Diagnostic sera from patients with confirmed infectious diseases other than MPX (VZV: varicella zoster virus; HSV: herpes simplex virus; Ent: enterovirus; CMV: cytomegalovirus; EBV: Epstein-Barr virus; HIV: human immunodeficiency virus) were selected and added to plates coated with a VACV-infected cell lysate at a serum concentration of 1:100. Sera from MVA-H5-vaccinated participants obtained 4 weeks after booster vaccination were included as positive control. (**c**) Several sera from all four cohorts were selected as ‘bridging’ samples and were measured in at least two independent ELISAs with VACV-infected cell lysate. Assays were correlated by performing Spearman r analysis. (**d**) A reference serum (either a pool of MVA-H5-vaccinated individuals, or a pool of historically-vaccinated and Imvanex-boosted individuals [P/B]) was included on every ELISA plate.

**Extended Data Fig. 2:**
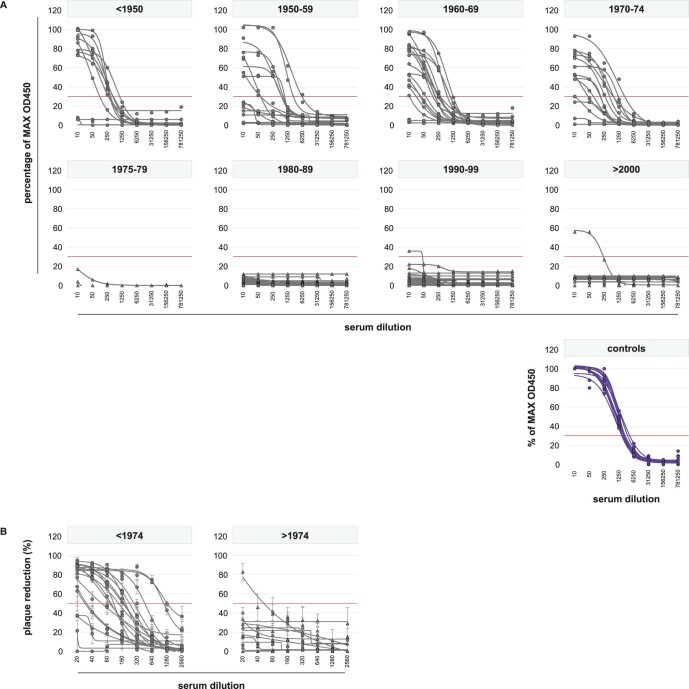
Normalized S-curves for determining the 30% OD450 ELISA endpoint and PRNT50 in age-panel sera. (**a**) Sera were 5-fold diluted starting at a dilution of 1:10 and added to plates coated with a VACV-infected cell lysate. A positive reference control was included on every plate to generate an S-curve with minimum (0%) and maximum (100%) OD450 values. All sample OD450 values were normalized to the positive control on the respective plate and plotted as log (inhibitor) vs response curve with four parameter variable slope using GraphPad Prism 9.02. A 30% endpoint dilution was estimated by calculating the proportionate distance between two dilutions, from which a titer was determined. 30% endpoint titers are shown in Fig. 1a. (**b**) Sera were 2-fold diluted starting at a dilution of 1:10 in duplicate, followed by the addition of virus suspension leading to ± 500 plaques/well in infection controls. Percentage plaque reduction compared to the infection control was calculated per dilution and plotted as log (inhibitor) vs response curve with four parameter variable slope using GraphPad Prism 9.02. The dilution that would yield 50% reduction of plaques compared to the infection control was estimated by determining the proportionate distance between two dilutions, from which an endpoint titer was calculated. PRNT50 values are shown in Fig. 1b. Symbols indicate individual donors, gray lines represent samples from the age-panel, purple lines represent the controls from the different ELISA plates. All samples from this serum panel were run in the same ELISA or PRNT experiment, avoiding any possible batch effects.

Immunogenicity of orthopoxvirus vaccines is generally measured via the presence of VACV-specific antibodies. Preliminary ELISA results based on the use of both VACV Elstree-infected and MVA-infected cell lysates highlighted a higher sensitivity of the ELISA performed with VACV Elstree-infected cell lysate (Extended Data Fig. 1a). To determine whether we could detect VACV-reactive immunoglobulin G (IgG) antibodies induced by historic smallpox vaccination, we first performed an ELISA with VACV Elstree-infected cell lysate and sera selected from the Erasmus Medical Center serum bank based on year of birth and divided over decades before or during 1974 (*n* = 59) and after 1974 (*n* = 67) (smallpox vaccination of the general population was stopped in 1974 in the Netherlands). The sera were all obtained in 2022, meaning that the sera from individuals born before 1950 were >70 years post-historic smallpox vaccination (the median number of years for this group between birth and sample was 74 years; range 72–85 years). VACV-reactive antibodies were frequently detected in the sera obtained from individuals born before 1974 (before or during 1974 = 71% (42/59); before 1950 = 73% (8/11); 1950–1959 = 53% (8/15) ; 1960–1969 = 84% (16/19); and 1970–1974 = 71% (10/14)) but infrequently in individuals born after 1974 (3% (2/67)) (Fig. 1a, Table 1 and Extended Data Fig. 2a; for before or during 1974 versus after 1974, *P* < 0.0001; Mann–Whitney *U*-test). From the sera tested by ELISA, we randomly selected 30 sera (*n* = 19 and 11 from individuals born before or during 1974 and after 1974, respectively; colored symbols in Fig. 1a) to assess the presence or absence of antibodies capable of neutralizing MPXV. Neutralization of MPXV was almost exclusively detected in the selection of sera from individuals born before 1974 (Fig. 1b and Extended Data Fig. 2b). Sera not capable of neutralizing MPXV were also negative for VACV-reactive antibodies in the ELISA.

**Table 1:** Overview of age panel sera assessed for the presence of VACV-reactive or MPXV-reactive antibodies by ELISA and MVA-neutralizing or MPXV-neutralizing antibodies by PRNT

		Before or during 1974	After 1974	Before 1950	1950–1959	1960–1969	1970–1974	1975–1979	1980–1989	1990–1999	After 2000
VACV ELISA											
Sera		59	67	11	15	19	14	4	25	27	11
Responders		42	2	8	8	16	10	0	0	1	1
Percentage (%)		71	3	73	53	84	71	0	0	4	9
GMT		115	11	152	93	112	121	10	10	10	13
MPXV ELISA											
Sera		19	11	2	4	8	5	–	4	7	–
Responders		8	1	1	2	3	2	–	0	1	–
Percentage (%)		42	9	50	50	38	40	–	0	14	–
GMT		16	10	14	24	13	18	–	10	10	–
MVA PRNT											
Sera		19	11	2	4	8	5	–	4	7	–
Responders		5	0	0	1	2	2	–	0	0	–
Percentage (%)		26	0	0	25	25	40	–	0	0	–
GMT		16	10	10	15	14	22	–	10	10	–
MPXV PRNT											
Sera		19	11	2	4	8	5	–	4	7	–
Responders		15	1	2	3	5	5	–	1	0	–
Percentage (%)		79	9	100	75	63	100	–	25	0	–
GMT		90	12	126	48	71	191	–	16	10	–
Total											
Sex (M/F)^a^		30/29	37/30	6/5	9/6	9/10	6/8	1/3	13/12	15/12	8/3
Age (years)^b^		61 (range, 49–85)	30 (range, 15–47)	74 (range, 72–85)	68 (range, 63–72)	58 (range, 52–62)	51 (range, 49–52)	46 (range, 43–47)	38 (range, 32–42)	29 (range, 23–32)	17 (range, 15–21)

^a^Number of assessed serum samples per group from males (M) versus females (F).

^b^The median age per group, including the upper and lower limit, is given.

GMT, geometric mean titer.

### MPXV infection induces or boosts VACV-reactive antibodies

**Extended Data Fig. 3:**
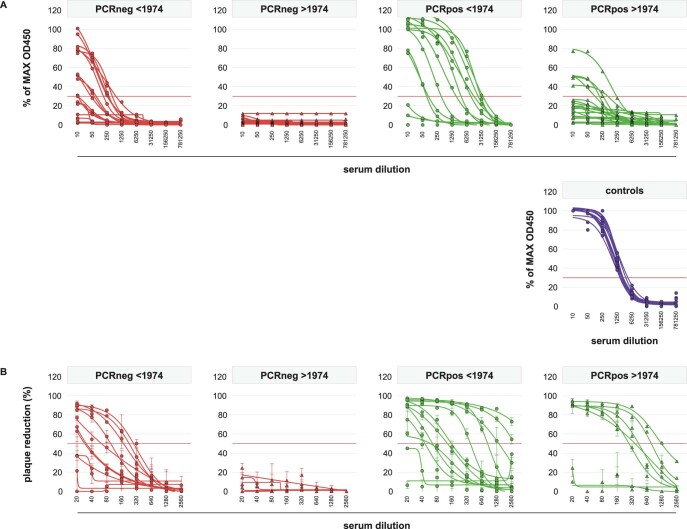
Normalized S-curves for determining the 30% OD450 ELISA endpoint and PRNT50 in diagnostic sera. (**a**) Sera were 5-fold diluted starting at a dilution of 1:10 and added to plates coated with a VACV-infected cell lysate. A positive reference control was included on every plate to generate an S-curve with minimum (0%) and maximum (100%) OD450 values. All sample OD450 values were normalized to the positive control on the respective plate and plotted as log (inhibitor) vs response curve with four parameter variable slope using GraphPad Prism 9.02. A 30% endpoint dilution was estimated by calculating the proportionate distance between two dilutions, from which a titer was determined. 30% endpoint titers are shown in Fig. 1c. (**b**) Sera were 2-fold diluted starting at a dilution of 1:10 in duplicate, followed by the addition of virus suspension leading to ± 500 plaques/well in infection controls. Percentage plaque reduction compared to the infection control was calculated per dilution and plotted as log (inhibitor) vs response curve with four parameter variable slope using GraphPad Prism 9.02. The dilution that would yield 50% reduction of plaques compared to the infection control was estimated by determining the proportionate distance between two dilutions, from which an endpoint titer was calculated. PRNT50 values are shown in Fig. 1d. Symbols indicate individual donors (circle: born prior to 1974, triangle born after 1974), red lines represent sera from PCR-negative donors, green lines represent sera from PCR-positive donors, and purple lines represent the controls from the different ELISA plates. All samples from this serum panel were run in the same ELISA or PRNT experiment, avoiding any possible batch effects.

To determine whether MPXV infection leads to the production of VACV-reactive IgG antibodies, we performed an ELISA with VACV Elstree-infected cell lysate on diagnostic sera submitted to our laboratory for MPXV quantitative polymerase chain reaction (PCR). We included the sera from individuals who tested negative for MPXV by PCR (*n* = 40, of which *n* = 19 sera were from individuals born before or during 1974) and positive for MPXV by PCR (*n* = 32, of which *n* = 13 sera were from individuals born before or during 1974) (Table 2). In MPXV PCR-negative individuals, VACV-reactive IgG antibodies were exclusively detected in participants born before or during 1974 (*n* = 10/19; 53%), reflecting antibodies induced by inferred historic smallpox vaccination. In MPXV PCR-positive individuals, from whom sera were exclusively obtained in the early symptomatic phase, VACV-reactive IgG antibodies were detected in individuals born before or during 1974 (*n* = 10/13; 77%) and after 1974 (*n* = 5/19; 26%) (Fig. 1c, Table 2 and Extended Data Fig. 3a). Because we had thus far infrequently detected VACV-reactive IgG antibodies in individuals born after 1974, we assume that these were induced by MPXV infection. Antibody responses were more frequently detected in MPXV PCR-positive individuals born before or during 1974, and the geometric mean antibody level was also significantly higher compared to inferred historic vaccination alone (*P* = 0.0082; Mann–Whitney *U*-test) or MPXV infection alone (*P* = 0.0010; Mann–Whitney *U*-test), suggestive of a rapid recall antibody response induced by MPXV infection (Fig. 1c, green symbols).

**Table 2:** Overview of diagnostic panel sera assessed for the presence of VACV-reactive or MPXV-reactive antibodies by ELISA and MVA-neutralizing or MPXV-neutralizing antibodies by PRNT

		Before 1974 and PCR^−^	Before 1974 and PCR^+^	After 1974 and PCR^−^	After 1974 and PCR^+^
VACV ELISA					
Sera		19	13	21	19
Responder		10	10	0	5
Percentage (%)		53	77	0	26
GMT		43	846	10	24
MPXV ELISA					
Sera		11	12	5	7
Responder		1	8	0	0
Percentage (%)		9	67	0	0
GMT		10	123	10	10
MVA PRNT					
Sera		11	12	5	7
Responder		0	5	0	0
Percentage (%)		0	42	0	0
GMT		10	41	10	10
MPXV PRNT					
Sera		11	12	5	7
Responder		7	10	0	5
Percentage (%)		64	83	0	71
GMT		41	185	10	176
Total					
Sex (M/F)^a^		14/5	12/1	17/4	19/0
Age (years)^b^		57 (range, 51–80)	57 (range, 52–65)	29 (range, 20–42)	35 (range, 21–41)

^a^Number of assessed serum samples per group from males (M) versus females (F).

^b^The median age per group, including the upper and lower limit, is given.

### Antibodies induced or boosted by MPXV infection neutralize MPXV

We randomly selected 35 sera from MPXV PCR-negative individuals (*n* = 16, of which *n* = 11 sera were from individuals born before or during 1974) and MPXV PCR-positive individuals (*n* = 19, of which *n* = 12 sera were from individuals born before or during 1974) to assess the presence of antibodies capable of neutralizing MPXV (colored symbols in Fig. 1c). Similar to the ELISA results, virus-neutralizing activity in sera from PCR-negative individuals was observed only in individuals born before or during 1974, probably reflective of antibodies induced by historic smallpox vaccination (Fig. 1d (red symbols) and Extended Data Fig. 3b). MPXV-neutralizing antibodies were also detected in sera from MPXV PCR-positive individuals born after 1974 (Fig. 1d (green symbols) and Extended Data Fig. 3b). Interestingly, the rapid boosting of VACV-reactive IgG antibodies after MPXV infection of individuals born before or during 1974 was not as obvious with respect to MPXV-neutralizing antibodies, as no significant differences were observed (Fig. 1d). However, a trend toward higher antibody levels in MPXV PCR-positive individuals born before or during 1974 was observed when compared with inferred historic vaccination by itself. When performing a direct comparison between the VACV-reactive IgG antibodies and MPXV-neutralizing antibodies, a good correlation was observed between VACV ELISA and MPXV 50% plaque reduction neutralization test (PRNT_50_) titers (Spearman’s correlation; *r* = 0.8325; *P* < 0.0001), with the exception of a cluster of sera obtained from exclusively MPXV-infected individuals (Fig. 1e, green triangles).

### Imvanex vaccination induces VACV-reactive IgG antibodies

**Fig. 2:**
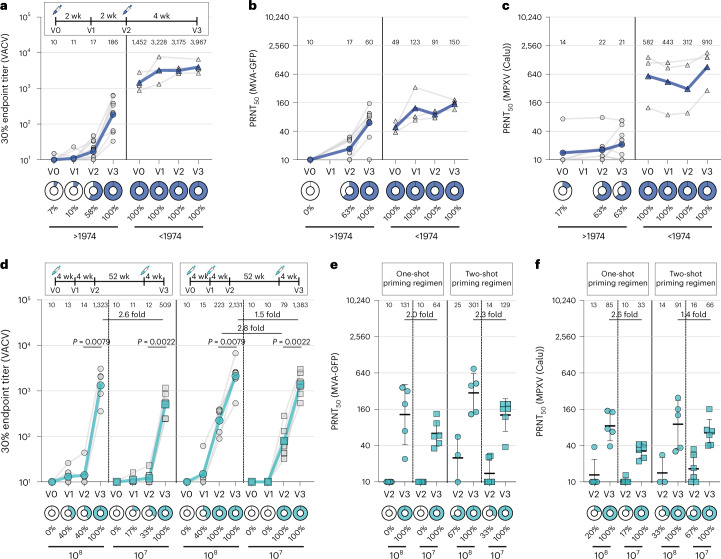
VACV-reactive and MPXV-neutralizing antibodies after Imvanex or MVA-H5 vaccination. **a**–**c**, Detection of poxvirus-specific antibodies in a panel of *n* = 56 sera obtained from *n* = 18 Imvanex-vaccinated participants. Sera were obtained pre-vaccination and at three timepoints post-vaccination from individuals born before or during 1974 (triangles) or after 1974 (circles). **a**, Detection of VACV-reactive antibodies by ELISA with VACV-Elstree-infected cell lysate. Gray symbols are data points, the colored symbols and line reflect the geometric mean. **b**, Detection of MVA-neutralizing antibodies by PRNT in a serum selection of *n* = 33 sera. Gray symbols are data points; the colored symbols and line reflect the geometric mean. **c**, Detection of MPXV-neutralizing antibodies by PRNT on a serum selection of *n* = 33 sera. Graey symbols are data points; the colored symbols and line reflect the geometric mean. **d**, Detection of VACV-reactive antibodies by ELISA with VACV Elstree-infected cell lysate. A total of *n* = 86 sera were obtained from *n* = 22 participants in an MVA-H5 vaccination trial who received either a high (10^8^) or low (10^7^) dose regimen, with two (left) or three (right) vaccinations. Vaccination regimens are indicated in the legend above the panel. Gray symbols are data points; the colored symbols and line reflect the geometric mean. Fold differences between geometric mean titers after the second or booster vaccination with 10^8^ or 10^7^ PFU are indicated. **e**, Detection of MVA-neutralizing antibodies by PRNT in a serum selection of *n* = 42 sera obtained at V2 or V3. Lines indicate geometric mean; whiskers indicate 95% confidence interval. Fold differences between geometric mean titers after the booster vaccination are indicated. **f**, Detection of MPXV-neutralizing antibodies by PRNT, similar to **e**. 30% endpoint ELISA titers were calculated based on a five-fold dilution series, after subtraction of OD_450_ values against a mock-infected cell lysate and relative to a positive control. The PRNT_50_ titer was calculated on the basis of a two-fold dilution series relative to an infection control. A Mann–Whitney *U*-test was performed to compare VACV-reactive endpoint titers at V2 and V3 in **d** (two-tailed *P* = 0.05 considered significant). wk, weeks.

**Extended Data Fig. 4:**
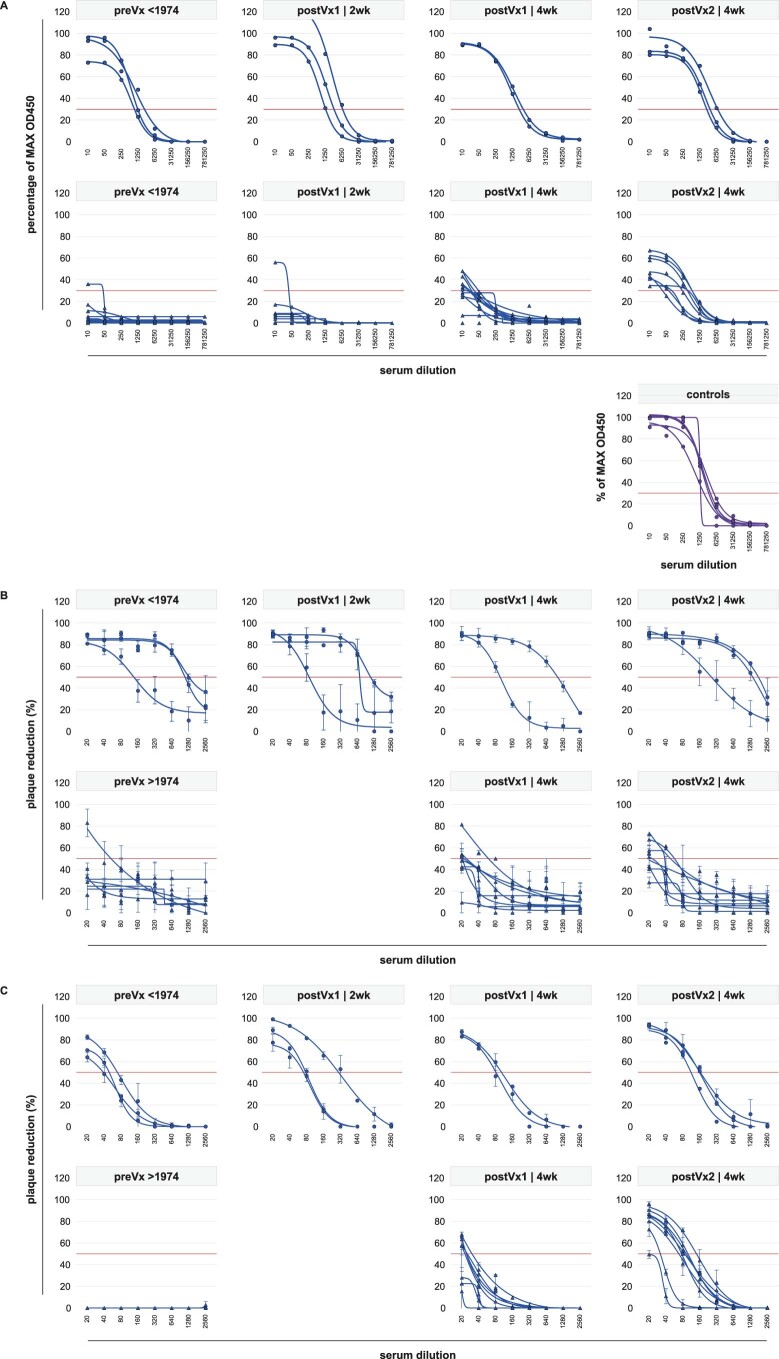
Normalized S-curves for determining the 30% OD450 ELISA endpoint and PRNT50 in Imvanex sera. (**a**) Sera were 5-fold diluted starting at a dilution of 1:10 and added to plates coated with a VACV-infected cell lysate. A positive reference control was included on every plate to generate an S-curve with minimum (0%) and maximum (100%) OD450 values. All sample OD450 values were normalized to the positive control on the respective plate and plotted as log (inhibitor) vs response curve with four parameter variable slope using GraphPad Prism 9.02. A 30% endpoint dilution was estimated by calculating the proportionate distance between two dilutions, from which a titer was determined. 30% endpoint titers are shown in Fig. 2a. (**b, c**) Sera were 2-fold diluted starting at a dilution of 1:10 in duplicate, followed by the addition of rMVA-GFP suspension (**b**) or MPXV suspension (**c**) leading to ± 500 plaques/well in infection controls. Percentage plaque reduction compared to the infection control was calculated per dilution and plotted as log (inhibitor) vs response curve with four parameter variable slope using GraphPad Prism 9.02. The dilution that would yield 50% reduction of plaques compared to the infection control was estimated by determining the proportionate distance between two dilutions, from which an endpoint titer was calculated. PRNT50 values are shown in Fig. 2b for rMVA-GFP and Fig. 2c for MPXV. Symbols indicate individual donors (circle: born prior to 1974, triangle born after 1974), blue lines represent sera from Imvanex-vaccinated donors, purple lines represent the controls from the different ELISA plates. All samples from this serum panel were run in the same ELISA or PRNT experiment, avoiding any possible batch effects.

To determine whether Imvanex vaccination leads to the production of VACV-reactive IgG antibodies, we performed an ELISA with VACV Elstree-infected cell lysates and sera obtained pre-vaccination (V0), 2 weeks and 4 weeks after the first vaccination (V1 and V2, respectively) and 4 weeks after the second vaccination (V3) (Fig. 2a, top). The serum samples were collected from healthcare workers who received Imvanex vaccination for safety reasons as employees of a Biosafety Level 3 (BSL-3) laboratory under the Erasmus Medical Center vaccination cohort (COVA) biobanking study protocol. Participants were vaccinated with the advised dose, 0.5 ml with no less than 5 × 10^7^ plaque-forming units (PFU). A total of 18 study participants were included (*n* = 3 born before or during 1974), of whom 11 were followed until the last timepoint at the time of writing (Table 3 and Extended Data Fig. 4a). VACV-reactive IgG antibodies were detected in all sera from study participants born before or during 1974 at all timepoints. A clear boosting by vaccination was not observed in these three individuals who already had high binding antibody levels before vaccination (Fig. 2a (right panel) compared with Fig. 1a). In study participants born after 1974, a gradual increase in binding antibody responses was seen, with detectable antibodies in 1/10 (10%) sera obtained 2 weeks after the first vaccination, 7/12 (58%) sera obtained 4 weeks after the first vaccination and 8/8 (100%) sera obtained 4 weeks after the second vaccination (Table 3 and Fig. 2a).

**Table 3:** Overview of Imvanex panel sera assessed for the presence of VACV-reactive or MPXV-reactive antibodies by ELISA and MVA-neutralizing or MPXV-neutralizing antibodies by PRNT. The timepoints V0 (pre-vaccination), V1 (two weeks after the first vaccination), V2 (four weeks after the first vacciantion) and V3 (four weeks after the second vaccination) refer to Fig. 2a

		Before 1974				After 1974			
		V0	V1	V2	V3	V0	V1	V2	V3
VACV ELISA									
Sera		3	3	2	3	15	10	12	8
Responder		3	3	2	3	1	1	7	8
Percentage (%)		100	100	100	100	7	10	58	100
GMT		1,452	3,228	3,175	3,967	10	11	17	186
MPXV ELISA									
Sera		3	3	2	3	6	–	8	8
Responder		2	3	2	3	1	–	0	0
Percentage (%)		67	100	100	100	17	–	0	0
GMT		24	110	50	96	10	–	10	10
MVA PRNT									
Sera		3	3	2	3	6	–	8	8
Responder		3	3	2	3	0	–	5	8
Percentage (%)		100	100	100	100	0	–	63	100
GMT		49	123	91	150	10	–	17	60
MPXV PRNT									
Sera		3	3	2	3	6	–	8	8
Responder		3	3	2	3	1	–	5	5
Percentage (%)		100	100	100	100	17	–	63	63
GMT		582	443	312	910	14	–	16	21
Total									
Sex (M/F)^a^		1/2				8/10			
Age (years)^b^		52 (range, 51–62)				30 (range, 24–45)			

^a^Number of assessed serum samples per group from males (M) versus females (F).

^b^The median age per group, including the upper and lower limit, is given.

### Imvanex induces low levels of MPXV-neutralizing antibodies

Thus far, MVA-BN immunogenicity has been assessed only by measuring MVA-specific and VACV-specific antibodies. Consequently, in addition to performing an ELISA with a VACV Elstree-infected cell lysate and an MVA neutralization assay, an MPXV neutralization assay was performed with sera from Imvanex-vaccinated study participants. Both MVA-neutralizing and MPXV-neutralizing antibodies were detected in study participants born before or during 1974 at all timepoints, including before Imvanex vaccination (Fig. 2b,c, right panels). Similarly to VACV-reactive binding antibodies, MVA-neutralizing antibodies were induced by vaccination and increased over time in participants born after 1974 (Extended Data Fig. 4b). Pre-vaccination, 0/6 (0%) sera from these individuals had detectable MVA-neutralizing antibodies, increasing to 5/8 (63%) and 8/8 (100%) after the first and second vaccination, respectively (Fig. 2b, left panel). In contrast, MPXV-neutralizing antibodies after vaccination with Imvanex were detected less frequently. Only in 5/8 (63%) sera were MPXV-neutralizing antibodies detected 4 weeks after the first and second vaccinations. Antibody levels in some vaccines increased after the second shot, but, in general, little increase in MPXV neutralization was observed after the second dose (Fig. 2c (left panel) and Extended Data Fig. 4c).

### A third MVA vaccination boosts antibody responses

**Extended Data Fig. 5:**
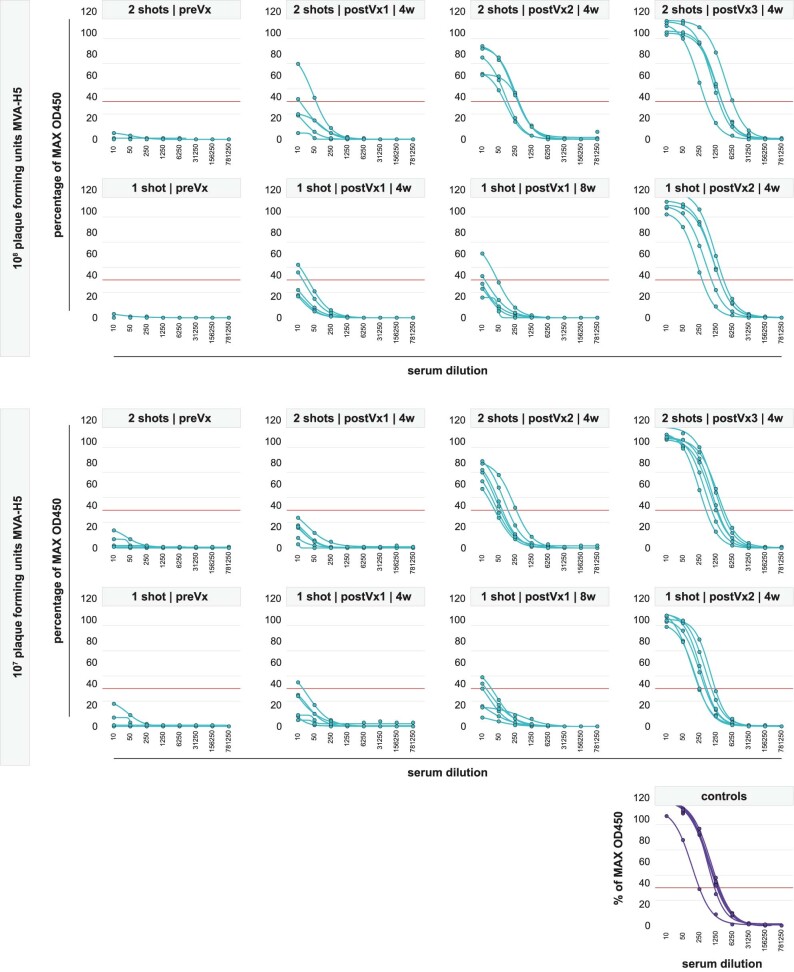
Normalized S-curves for determining the 30% OD450 ELISA endpoint in MVA-H5 sera. Sera from the MVA-H5 panel were 5-fold diluted starting at a dilution of 1:10 and added to plates coated with a VACV-infected cell lysate. A positive reference control was included on every plate to generate an S-curve with minimum (0%) and maximum (100%) OD450 values. All sample OD450 values were normalized to the positive control on the respective plate and plotted as log (inhibitor) vs response curve with four parameter variable slope using GraphPad Prism 9.02. A 30% endpoint dilution was estimated by calculating the proportionate distance between two dilutions, from which a titer was determined. Symbols indicate individual donors, cyan lines represent sera from MVA-H5-vaccinated donors, purple lines represent the controls from the different plates. 30% endpoint titers are shown in Fig. 2d. All samples from this serum panel were run in the same experiment, avoiding any possible batch effects.

**Extended Data Fig. 6:**
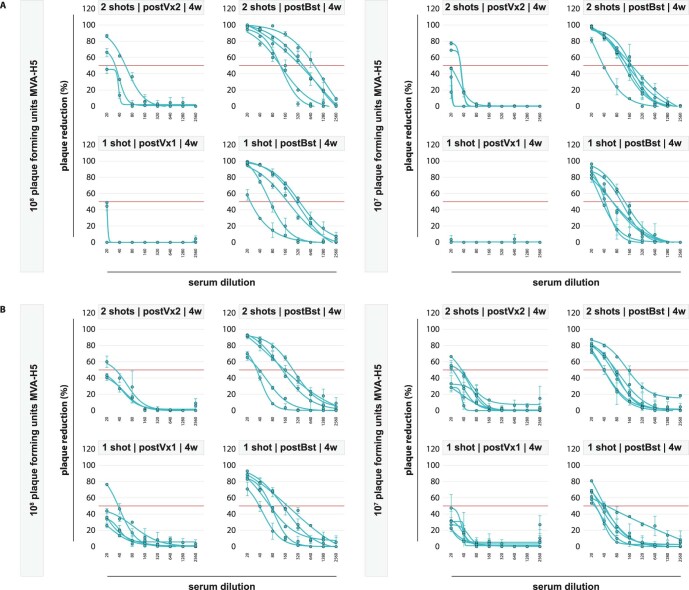
Normalized S-curves for determining the PRNT50 MVA-H5 sera. (**a**) Sera were 2-fold diluted starting at a dilution of 1:10 in duplicate, followed by the addition of rMVA-GFP suspension (**a**) or MPXV suspension (**b**) leading to ± 500 plaques/well in infection controls. Percentage plaque reduction compared to the infection control was calculated per dilution and plotted as log (inhibitor) vs response curve with four parameter variable slope using GraphPad Prism 9.02. The dilution that would yield 50% reduction of plaques compared to the infection control was estimated by determining the proportionate distance between two dilutions, from which an endpoint titer was calculated. PRNT50 values are shown in Fig. 2e for MVA-GFP and Fig. 2f for MPXV. Symbols indicate individual donors, cyan lines represent sera from MVA-H5-vaccinated donors. All samples from this serum panel were run in the same ELISA or PRNT experiment, avoiding any possible batch effects.

To further assess MVA immunogenicity, we performed an ELISA with a lysate of VACV Elstree-infected cells and sera from an MVA-H5 influenza vaccination trial^13^. Sera were obtained from study participants (who were all born after 1974) who received vaccinations following two different regimens: (1) a single-shot primary vaccination regimen followed by a boost after 1 year or (2) a two-shot primary vaccination regimen followed by a boost after 1 year. Additionally, participants under each regimen received either a high (10^8^ PFU) or low (10^7^ PFU) dose of MVA-H5. Sera were obtained 4 weeks after each vaccination or 8 weeks in the case of the primary vaccination series for the single-shot vaccination regimen (Fig. 2d, top). We observed similar levels of VACV-reactive antibodies 4 weeks after two shots of Imvanex or MVA-H5 (compare 10^8^ PFU MVA-H5 4 weeks after the second vaccination (V2; Fig. 2d) with Imvanex 4 weeks after the second vaccination (V3; Fig. 2a) (*P* = 0.8329; Mann–Whitney *U*-test) and compare 10^7^ PFU MVA-H5 4 weeks after the second vaccination (V2; Fig. 2d) with Imvanex 4 weeks after the second vaccination (V3; Fig. 2a) (*P* = 0.1812; Mann–Whitney *U*-test)). The ELISAs with VACV Elstree-infected lysates for the Imvanex-vaccinated and MVA-H5-vaccinated cohorts were performed separately, but a bridging reference sample was included on every assay plate. In comparing different dosing and vaccine regimens, we found that vaccination with a high dose resulted in higher antibody titers; 2.8-fold higher binding antibody levels were elicited by two shots of 10^8^ PFU compared with two shots of 10^7^ PFU, and 1.5-fold and 2.6-fold higher binding antibody levels were found when comparing the booster vaccination after the two-shot versus one-shot primary regimen, respectively (Extended Data Fig. 5 and Fig. 2d). The second vaccination appeared to be crucial for reaching detectable antibody levels, as individuals under the single-shot regimen developed no to low antibody responses 4 weeks and 8 weeks after vaccination. Finally, a booster vaccination given after 1 year boosted the binding antibody levels in all dosing groups (up to 18-fold in the two-shot regimen and over 40-fold for both doses (10^8^ and 10^7^ PFU) in the one-shot regimen) (Table 4 and Fig. 2d). Additionally, MVA and MPXV neutralization assays were performed with 42 sera obtained 4 weeks (two-shot regimen) or 8 weeks (one-shot regimen) after the second or first shot, respectively (V2) and 4 weeks after the booster vaccination (V3) (Extended Data Fig. 6). Similarly to two shots of Imvanex, we observed low levels of neutralizing antibodies against both MVA (Fig. 2e) and MPXV (Fig. 2f) after two shots of MVA-H5. A third booster vaccination significantly increased neutralizing antibody levels and elevated seropositivity rates for both MVA and MPXV to 100%, independent of the dosing or vaccination regimen. Simultaneously, geometric mean MPXV-neutralizing antibody levels of the high-dose regimens were 1.4-fold and 2.6-fold higher compared to the low-dose regimen for the two-shot and one-shot primary regimens, respectively.

**Table 4:** Overview of MVA-H5 panel sera assessed for the presence of VACV-reactive or MPXV-reactive antibodies by ELISA and MVA-neutralizing or MPXV-neutralizing antibodies by PRNT. The timepoints V0 (pre-vaccination), V1 (four weeks after the first vaccination), V2 (four/eight weeks after the first/second vaccination, respectively) and V3 (four weeks after the third vaccination) refer to Fig. 2d

		One shot				Two shots			
		V0	V1	V2	V3	V0	V1	V2	V3
VACV ELISA (10^7^ PFU)									
Sera		6	6	6	6	6	6	6	6
Responder		0	1	2	6	0	0	6	6
Percentage (%)		0	17	33	100	0	0	100	100
GMT		10	11	12	509	10	10	79	1,383
MVA PRNT (10^7^ PFU)									
Sera		–	–	6	6	–	–	6	6
Responder		–	–	0	6	–	–	2	6
Percentage (%)		–	–	0	100	–	–	33	100
GMT		–	–	10	64	–	–	14	129
MPXV PRNT (10^7^ PFU)									
Sera		–	–	6	6	–	–	6	6
Responder		–	–	1	6	–	–	4	6
Percentage (%)		–	–	17	100	–	–	67	100
GMT		–	–	10	33	–	–	16	66
VACV ELISA (10^8^ PFU)									
Sera		5	5	5	5	3	5	5	5
Responder		0	2	2	5	0	2	5	5
Percentage (%)		0	40	40	100	10	40	100	100
GMT		10	13	14	1,323	10	15	223	2,131
MVA PRNT (10^8^ PFU)									
Sera		–	–	5	5	–	–	3	5
Responder		–	–	0	5	–	–	2	5
Percentage (%)		–	–	0	100	–	–	67	100
GMT		–	–	10	131	–	–	25	301
MPXV PRNT (10^8^ PFU)									
Sera		–	–	5	5	–	–	3	5
Responder		–	–	1	5	–	–	1	5
Percentage (%)		–	–	20	100	–	–	33	100
GMT		–	–	13	85	–	–	14	91
Total									
Sex (M/F)^a^		NA				NA			
Age (years)^a^		NA (range, 18–28)				NA (range, 18–28)			

^a^The original study included both male and female volunteers between 18 and 28 years of age. Exact allocation of sex and age to the selected samples was not possible here.

### Discrimination between MVA vaccination and MPXV infection

**Extended Data Fig. 7:**
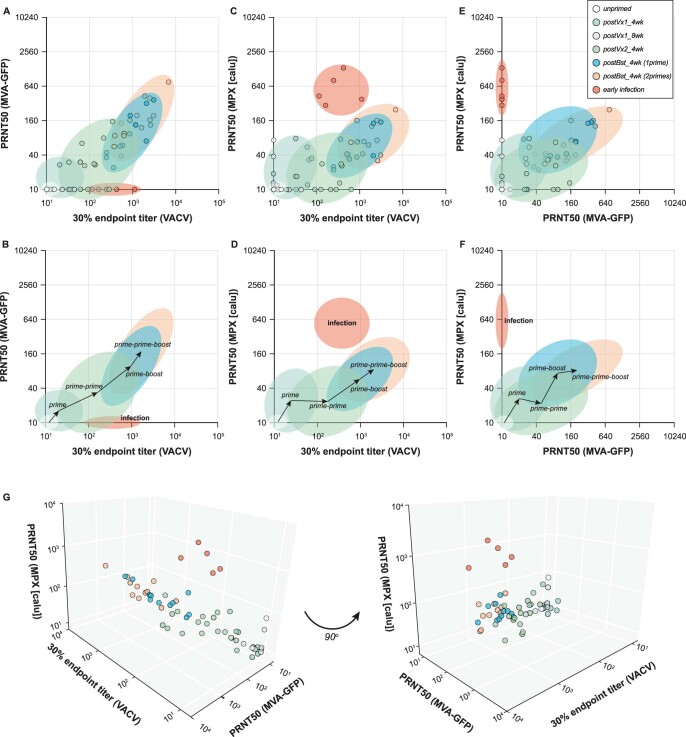
Differentiation between MVA vaccination and MPXV infection by serology. ELISAs were performed on sera from either vaccinated and MPXV PCR-positive individuals with a VACV-infected cell lysate. 30% endpoint ELISA titers were calculated based on a 5-fold dilution series, after subtraction of OD450 values with a mock-infected cell lysate, and relative to a positive control. PRNTs were performed with MVA or MPXV. The 50% plaque reduction neutralization titer (PRNT50) was calculated on the basis of a 2-fold dilution series relative to an infection control. (**a, b**) Correlation of VACV ELISA and MVA PRNT. (**c, d**) Correlation of VACV ELISA and MPX PRNT. (**e, f**) Correlation of MVA PRNT and MPXV PRNT. The bubbles in panels A, C, and E enclose all datapoints from the indicated samples set. The centers of bubbles from vaccinated individuals were connected by arrows to indicate antibody level increases, as indicated in panels B, D, and F. (G) 3D representation of the data presented in panels A, C, and E.

As shown earlier, both MVA vaccination and MPXV infection alone result in the induction of VACV-reactive antibodies (Figs. 1c and 2a,d). Consequently, using only an ELISA with VACV Elstree-infected cell lysates does not allow serological differentiation between MPXV infection and MVA vaccination in affected individuals. However, this differentiation can be of crucial importance in serosurveys and/or diagnostics in the general population and particularly at-risk groups. To determine whether a combination of the assays employed above allows serological differentiation of MVA vaccination from MPXV infection, we performed correlations between the ELISA with a VACV Elstree-infected cell lysate and the PRNTs with infectious MVA and MPXV. Sera from both MPXV PCR-positive patients and Imvanex-vaccinated and MVA-H5-vaccinated individuals were included in this analysis. The sera from the MPXV PCR-positive patients could be serologically distinguished from sera obtained from vaccinated individuals by a combination of high MPXV-neutralizing antibody levels and absent MVA-neutralizing antibodies, suggesting limited cross-neutralization of sera obtained from MVA-vaccinated and MPXV-infected individuals (Extended Data Fig. 7a,c,e). As for sera from vaccinated individuals, a sequential increase in antibodies detected in all assays was observed in individuals receiving one priming vaccination, two priming vaccinations, a priming vaccination followed by a booster or two priming vaccinations followed by a booster (Extended Data Fig. 7b,d,f). A three-dimensional representation of the data showed clear separate clustering of the MPXV PCR-positive sera (Extended Data Fig. 7g).

### Validation of assays detecting MPXV(-neutralizing) antibodies

**Extended Data Fig. 8:**
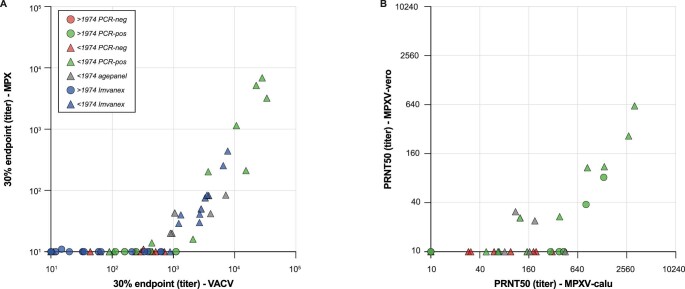
Correlation between ELISAs with VACV and MPXV antigen, and neutralization assays with MPXV grown on Vero or Calu-3 cells. (**a**) ELISAs were performed on the age-panel, diagnostic panel, and Imvanex panel of sera with a VACV-infected or MPXV-infected cell lysate. 30% endpoint ELISA titers were calculated based on a 5-fold dilution series, after subtraction of the OD450 values from mock-infected cell lysate from the OD450 values from VACV-infected or MPXV-infected cell lysates, and relative to a positive control. (**b**) PRNTs were performed with an MPXV stock grown on either Calu-3 or Vero cells. The 50% plaque reduction neutralization titer (PRNT50) was calculated on the basis of a 2-fold dilution series relative to an infection control. Sera were obtained from individuals born in or prior to (triangles), or after (circles) 1974, who were either PCR-negative (red and gray), PCR-positive (green), or vaccinated (blue).

Thus far, we assessed the presence of binding and neutralizing antibodies in sera using an ELISA with a VACV Elstree-infected cell lysate and PRNTs with infectious MPXV grown on Calu-3 cells. Additionally, we developed an ELISA with an MPXV-infected cell lysate and directly compared the 30% endpoint titers obtained from either the VACV or MPXV ELISA for the age panel, diagnostic panel and Imvanex panel of sera (Extended Data Fig. 8a and original data in Figs. 1a,c and 2a). The ELISA with the MPXV-infected cell lysate appeared to be less sensitive and only detected antibodies if the 30% endpoint titer against VACV was >1,000. Additionally, we compared PRNT_50_ titers against infectious MPXV grown on either Calu-3 or Vero cells for the age panel and diagnostic panel of sera (Extended Data Fig. 8b). The PRNT against Vero-grown MPXV was considerably less sensitive, predominantly detecting neutralizing antibodies at PRNT_50_ values of >640 against Calu-3-grown MPXV.

## Discussion

In this study, we measured MVA-reactive, VACV-reactive and MPXV-reactive binding and neutralizing antibodies in cohorts of historic smallpox-vaccinated, MPXV PCR-positive, MVA-BN-vaccinated and MVA-H5-vaccinated individuals. For the development and validation of novel assays, an MPXV isolate obtained during the ongoing outbreak was used. We show that MPXV-neutralizing antibodies can be detected across individuals after MPXV infection, although we only detected MPXV-reactive antibodies in 5/19 MPXV-infected individuals born after 1974. We speculate that this was due to the sampling timepoint—in the early symptomatic phase. Additionally, we detected MPXV-neutralizing antibodies after historic smallpox vaccination. Strikingly, a two-shot MVA-BN immunization series in non-primed individuals yields relatively low antibody levels, with poor neutralizing capacity. Using sera from an MVA-H5 trial, we show that dose sparing leads to lower antibody levels than those elicited by a complete two-shot vaccination regimen, whereas a third MVA vaccination boosts both binding and neutralizing antibody responses. In summary, we show a relatively low neutralizing antibody response in sera from individuals double vaccinated with Imvanex.

Although little is known about the antigenic similarities among poxviruses, MVA-BN immunogenicity has thus far only been assessed by measuring MVA-specific and VACV-specific antibodies^5^, and cross-reactivity with other poxviruses is assumed. We argue that, for effective use of this vaccine during an ongoing MPXV outbreak, it is essential to measure the functionality of vaccine-induced antibodies against the currently circulating MPXV strain. To assess antigenic similarities among poxviruses and select the most appropriate serological assays for studying MPXV-reactive immune responses, we compared the binding and neutralizing activity of sera from infected and/or vaccinated individuals against VACV, MVA and/or MPXV. Measuring VACV-reactive binding antibodies by ELISA proved sensitive, as serological responses were detected in the majority of sera from participants born before 1974 and in the majority of recent vaccinees. No apparent waning in total binding antibody levels as a function of age was detectable in individuals born before 1974, supporting previous assertions about the longevity of antibodies induced by vaccinia-based smallpox vaccination^14,15^. However, because we did not have access to historic vaccination records, we could not confirm whether people born before 1974 were indeed vaccinated against smallpox, how many shots they received and which vaccine was used. Measuring MPXV-reactive binding antibodies with an in-house-developed ELISA proved less sensitive.

Because the currently used vaccine is based on MVA, proper assessment of vaccine immunogenicity should involve measuring both MVA-specific and MPXV cross-reactive neutralizing antibodies. Interestingly, only limited correlation was observed between MVA and MPXV neutralization in MVA-BN-vaccinated or MPXV-infected individuals, indicative of antigenic differences among these poxviruses. Depending on the research question, this suggests that measurement of a combination of both VACV-reactive antibodies and MVA- and MPXV-neutralizing antibodies may be required to study vaccine immunogenicity. By combining assays, it proved possible to serologically differentiate MVA vaccination from MPXV infection, which could be essential in future serosurveys among vaccinated risk groups.

The present study has some intrinsic limitations. It was designed as an immunogenicity study of MVA-BN with a focus on MPXV-reactive antibodies, as there was virtually no information on this in the published literature at the time of writing. This study was not intended to ascertain vaccine efficacy, which, in the Netherlands, is an ongoing effort based on national clinical data collection for all MPXV cases notified by sexually transmitted disease clinics. Although we were able to include four diverse groups of vaccinees and patients in our study, cohort sizes were inherently limited by the availability of samples. The pseudonymized serum samples from the diagnostic cohort were collected from patients with suspected MPXV infection, for whom our laboratory serves as a diagnostic center. The serum samples from the age panel cohort can be considered convenience samples, which were obtained from the serum bank at the Department of Viroscience, Erasmus Medical Center, and are thus unrelated to MPXV diagnostics. Both of these cohorts were fully anonymized in agreement with privacy legislation for retrospective studies based on the reuse of stored diagnostic samples. This provides the ability to rapidly assess essential assays and responses during an outbreak such as this but does not allow the linking of individual samples to clinical data, the course of infection or (historic) smallpox vaccination status. Finally, orthopoxviruses produce two major forms of infectious virions during their replication cycle: (1) IMVs and (2) extracellular enveloped virions (EEVs). IMVs are thought to be well-suited for transmission between hosts, whereas EEVs may have an important role in dissemination within the host^16^. The assays as employed in this study do not distinguish IMVs from EEVs, but plaque reduction assays generally measure the neutralization of IMVs rather than EEVs.

The evidence for cross-protection afforded by VACV or MVA vaccination against MPX is inferred from animal experiments and from observational studies conducted during the period of enhanced surveillance in the endgame of smallpox eradication^6–9^. In those studies, partial clinical protection was observed. In our study, the individuals born before 1974 still had detectable antibodies that neutralized MPXV, yet current epidemiological data suggest limited protection from infection in this age group (diagnostic observations). This is in line with earlier studies detecting subclinical MPXV infection in pre-immune individuals by serology^14^. The primary MVA immunization series in non-primed individuals yielded relatively low levels of neutralizing antibodies, raising the question of whether vaccinated individuals are now protected and what the correlates of protection against MPXV infection are. In non-human primates, depletion studies underlined that antibodies do play an important role against lethal intravenous MPXV challenge^17^, although both virus-specific antibodies and T cells were induced by MVA-BN vaccination. At this moment, it is unclear what the relatively low MPXV-neutralizing titers mean for protection against disease, severity of symptoms and transmissibility. Finally, using a serum set from a previously performed MVA-H5 clinical trial, we showed that VACV-reactive as well as MVA-neutralizing and MPXV-neutralizing antibodies can be further boosted with an additional shot of MVA. This same trial indicates that dose sparing (10^7^ instead of 10^8^ PFU) has a negative effect on the serological outcome of vaccination. Cohort studies following vaccinated individuals and including biological sampling are necessary to further assess vaccine efficacy in risk populations and to determine correlates of protection for this emerging pathogen.

## Methods

### Serum samples and ethics statement

The research presented here complies with all of the relevant ethical guidelines and was approved by the Erasmus Medical Center Medical Ethics Committee. Study protocols are mentioned below for the separate serum sets. Serum samples from *n* = 238 participants were included in this study, divided over four different cohorts (Tables 1–4 and Extended Data Figs. 2–6): (1) an age panel cohort; (2) a diagnostic cohort; (3) an Imvanex cohort; and (4) an MVA-H5 cohort. The age panel cohort was used to validate the assays and was retrieved from the diagnostic serum bank at Erasmus Medical Center, based on year of birth and excluding immunocompromised patients. In total, *n* = 126 sera collected in 2022 were included in this cohort (*n* = 59 born before or during 1974; *n* = 67 born after 1974). For the diagnostic cohort, diagnostic serum samples were collected in the Netherlands, in addition to swab samples for PCR testing from patients suspected of having MPXV infection. These were submitted to Erasmus Medical Center as a diagnostic center for MPX after privacy coding/pseudonymization by the respective sender. In total, *n* = 72 anonymized diagnostic sera were included in this study for further assay validation, subdivided into PCR-negative and PCR-positive patients, born either before or during 1974 or after 1974. Both the age panel and the diagnostic cohort were fully anonymized in agreement with privacy legislation for the retrospective studies based on the reuse of stored diagnostic samples. For the Imvanex cohort, serum samples were obtained from healthcare workers who received Imvanex vaccination for safety reasons as employees of a BSL-3 laboratory. Samples were collected under the Erasmus Medical Center vaccination cohort (COVA) biobanking study protocol (MEC-2014-398), and written informed consent was obtained from all participants. Longitudinal samples were obtained pre-vaccination, 2 weeks and 4 weeks after the first vaccination and 4 weeks after the second vaccination. Participants were vaccinated with the prescribed dose—0.5 ml with no fewer than 5 × 10^7^ PFU. A total of 18 participants were included (*n* = 3 born before or during 1974), of whom 11 were followed until the last timepoint at the time of writing. The fourth serum panel (MVA-H5 cohort) consisted of samples that were obtained from participants as part of a past clinical phase 1 vaccination trial with MVA-H5 in two different regimens (Fig. 2d), vaccinated with either a low dose (10^7^ PFU) or a high dose (10^8^ PFU)^13^. Longitudinal samples were obtained pre-vaccination, 4 weeks after the first vaccination, 4 weeks after the second vaccination or 8 weeks after the first vaccination (depending on the respective dosing regimen) and 4 weeks after the booster vaccination after 1 year. All study participants, independent of vaccination regimen or dosage, received a booster vaccination. The Erasmus Medical Center Medical Ethics Committee gave ethical approval for this work, which was performed as part of the FluVec-H5 study (ethical permit METC NL37002.000.12; Dutch Trial Registry NTR3401). Written informed consent was obtained from all participants. A total of 22 participants were included in this study, all born after 1974. Sex and gender were not considered in the study design. For cohorts (1) and (3), the study design included information on sex, and the numbers of males and females were comparable. Of the MPXV PCR-positive individuals in the diagnostic cohort, 97% were male, most of whom were men who have sex with men.

### Cell culture

CEFs were isolated from 11-day-old chicken embryos (Drost Loosdrecht BV) and passaged once before use. CEFs were cultured in virus production serum-free medium (VP-SFM, Gibco) containing penicillin and streptomycin. Baby hamster kidney 21 (BHK-21, American Type Culture Collection (ATCC)) cells were cultured in DMEM (Lonza) supplemented with 10% FBS, 20 mM HEPES, 0.1% CHNaO_3_, 0.1 mM non-essential amino acids (Lonza) and penicillin and streptomycin/l-glutamine. HeLa cells (ATCC) were cultured in DMEM supplemented with 10% FBS, 20 mM HEPES, 0.1% CHNaO_3_ and penicillin and streptomycin/l-glutamine. Vero cells (ATCC) were cultured in DMEM (Capricorn Scientific) supplemented with 10% FBS, 20 mM HEPES and penicillin and streptomycin/l-glutamine. Calu-3 cells (ATCC) were cultured in Opti-MEM + GlutaMAX (Gibco) supplemented with 10% FBS. All cell lines were grown at 37 °C in a humidified CO_2_ incubator.

### VACV Elstree virus and the generation of recombinant rMVA-GFP

Recombinant MVA expressing green fluorescent protein (rMVA-GFP) was generated by homologous recombination, as described previously^18^. MVA clonal isolate F6 served as the parental virus for generating rMVA-GFP^19^. Vector plasmid pG06-P11-GFP was used to direct the insertion of GFP under the transcriptional control of the natural VACV late promoter P11 into the deletion III site of the MVA genome. Virus stocks were generated in CEFs, purified by ultracentrifugation through 36% sucrose and reconstituted in a 120 mM NaCl and 10 mM Tris-HCl buffer (pH 7.4). The titer of the rMVA-GFP stock was initially determined by plaque assay on CEFs and was confirmed for PRNT by titration on Vero cells. The stock was validated by PCR, sequencing and transgene expression in various cell types. The VACV strain Elstree was a kind gift from K. J. Stittelaar^9^ and was grown in HeLa cells to serve as an ELISA antigen. All work with rMVA-GFP was performed in a Class II Biosafety Cabinet under BSL-2 conditions. Work with VACV was performed under BSL-2 conditions using BSL-3 safety measures (BSL-2+).

### Isolation and propagation of MPXV

MPXV was isolated from a swab taken from a typical pox lesion of an MPX-positive Dutch patient by inoculating Vero cells. The isolate belongs to clade IIB and was designated as MPXV_2022_NL001. It is available through the European Virus Archive Global (Ref-SKU: 010V-04721). Virus stocks were propagated to passage 3 by inoculating 70–90% confluent Vero and Calu-3 cultures grown in T175 flasks at a multiplicity of infection of 0.1 in Advanced DMEM/F-12 (Gibco) supplemented with 10 mM HEPES, 1× GlutaMAX and 1× primocin (AdDF+++). After 4 days, when at least 50% of the surface area in the cultures consisted of visible plaques, cells were harvested using a cell scraper and centrifuged at 2,000*g* for 2 minutes. Cell pellets were resuspended in 500 µl of Opti-MEM + GlutaMAX and pipetted up and down to mix ten times using a P1000 tip. Cell suspensions were lysed by freeze-thawing three times in a dry-ice ethanol bath, after which the lysates were mixed by pipetting up and down 50 times using a P1000 tip. To the lysates, we added 10 ml of Opti-MEM + GlutaMAX, which was then cleared by centrifugation at 2,000*g* for 5 minutes. Cleared lysates were filtered through a low-protein-binding 0.45-µm syringe filter (Millipore), aliquoted and frozen at −80 °C. All of the work with infectious MPXV was performed in a Class II Biosafety Cabinet under BSL-3 conditions.

### MPXV stock titrations

Stock titers were determined by preparing ten-fold serial dilutions in AdDF+++. We added 100 μl of each dilution to Vero cells in a 96-well plate and incubated them for 16 hours in a humidified CO_2_ incubator at 37 °C. Next, the cells were fixed in 10% neutral buffered formalin for 30 minutes and permeabilized in 70% ethanol (submerging the entire plate). Cells were then washed in PBS, blocked in 0.6% BSA (Sigma–Aldrich) and 0.1% Triton X-100 (Sigma–Aldrich) in PBS for 30 minutes and stained overnight at room temperature with rabbit-anti-VACV-FITC (Abbexa) at a 1:1,000 dilution. After washing in PBS, plates were scanned on the Amersham Typhoon Biomolecular Imager (channel = Cy2; resolution = 10 mm; GE Healthcare). The numbers of infected cells were quantified using ImageQuant TL 8.2 (GE Healthcare).

### Detection of VACV-specific or MPXV-specific IgG antibodies by ELISA

For the detection of VACV-specific or MPXV-specific antibodies, HeLa or Vero cells were mock treated or infected with VACV Elstree or MPXV_2022_NL001 at multiplicities of infection of 1 or 0.1, respectively, and harvested in 1% Triton X-100 in PBS supplemented with cOmplete Mini EDTA-free protease inhibitor (Roche) when a complete cytopathic effect was observed. All of the work with the MPXV lysate was performed under BSL-3 conditions. For ELISA, high-binding 96-well plates (Corning) were coated for 1 hour at 37 °C with the diluted cell lysates (VACV coating: 1:500; MPXV coating: 1:100) in PBS. The coating concentrations were optimized in coating titration experiments. Coated plates were washed five times with PBS supplemented with 0.05% Tween 20 (PBST; Merck) and subsequently blocked for 1 hour at 37 °C with blocking buffer (PBST + 2% skim milk powder (wt/vol, Merck)). Five-fold dilution series of sera in blocking buffer (starting dilution: 1:10) were transferred to the lysate-coated plates and incubated overnight at 4 °C. Plates were washed five times with PBST and incubated for 1 hour at 37 °C with horseradish peroxidase-conjugated goat-anti-human IgG at a dilution of 1:6,000 (Dako). Afterwards, the plates were again washed five times with PBST and incubated for about 15 minutes with 100 µl of TMB Peroxidase Substrate (SeraCare/KPL), after which the reaction was stopped with an equal volume of 0.5 N H_2_SO_4_ (Merck). The absorbance was measured at 450 nm using a Tecan Infinite F200 or Anthos 2001 microplate reader and corrected for absorbance at 620 nm. Values of optical density measured at a wavelength of 450 nm (OD_450_ values) were obtained with mock-infected cell lysates and subtracted from the OD_450_ value obtained with the VACV-/MPXV-infected cell lysates to determine a net OD_450_ response. A positive control serum was included on every ELISA plate, generating a minimum-to-maximum OD_450_ S-curve. OD_450_ values generated by dilution series per sample were transformed to this control S-curve, and 30% endpoint titers were calculated.

### Detection of MVA-/MPXV-specific neutralizing antibodies by PRNT

Vero cells were seeded 1 day before the experiment in 96-well plates (Greiner Bio-One) at a density of 20,000 cells per well. Sera were heat-inactivated for 1 hour at 60 °C and subsequently two-fold serially diluted in AdDF+++ before 1,500 PFU of rMVA-GFP or 400 PFU of MPXV_2022_NL001 in 60 μl was added per well. The final serum dilution in the first column was 1:20. The virus–serum mix was then incubated for 1 hour at 37 °C before 100 μl of it was added to the Vero cells. The cells were incubated for 24 hours (rMVA-GFP) or 16 hours (MPXV) at 37 °C and under 5% CO_2_ before fixing in 4% paraformaldehyde for 10 minutes (rMVA-GFP) or in 10% neutral buffered formalin for 30 minutes (MPXV). MPXV-infected samples were furthermore permeabilized in 70% ethanol, followed by a wash with PBS and blocking in 0.6% BSA and 0.1% Triton X-100 in PBS for 30 minutes before being stained overnight at room temperature with rabbit-anti-VACV-FITC (Abbexa) at a 1:1,000 dilution. Both MVA and MPXV neutralization assays were washed with PBS before nuclear staining with Hoechst 33342 (Thermo Fisher Scientific). Cells were imaged using the Opera Phenix spinning disk confocal HCS system (PerkinElmer) equipped with a ×10 air objective (NA 0.3) and 405-nm and 488-nm solid-state lasers. Hoechst and GFP/FITC were detected using 435–480-nm and 500–550-nm emission filters, respectively. Nine fields per well were imaged, covering approximately 50% of the individual wells. The number of infected cells was quantified using Harmony software (version 4.9, PerkinElmer). The dilution that would yield 50% reduction of plaques compared with the infection control was estimated by determining the proportionate distance between two dilutions from which an endpoint titer was calculated. When no neutralization was observed, the PRNT_50_ was given a value of 10.

### Data acquisition and statistical analysis

All samples from each respective experimental panel (that is, the age panel (1), the diagnostic panel (2), the Imvanex-vaccinated panel (3) and the MVA-H5-vaccinated panel (4)) were analyzed simultaneously per assay to counteract batch effects. ELISAs were thoroughly validated via three additional methods: (1) performing ELISAs with additional negative control sera obtained from patients diagnosed with other infectious diseases (Extended Data Fig. 1b); (2) side-by-side comparison of the results of bridging samples measured in two independent assays (Extended Data Fig. 1c); and (3) inclusion of the same reference serum on every ELISA plate (Extended Data Fig. 1d). For ELISAs with the VACV Elstree-infected cell lysate, the reference serum was a pool of two high-titer sera from individuals who received both historic smallpox and recent Imvanex vaccination. For ELISAs using MXPV-infected cell lysate, the reference serum was from an individual who received historic smallpox vaccination and had recent MPXV exposure. The reference samples on every plate were used to generate S-curves (Extended Data Figs. 2a, 3a, 4a and 5) to which the test samples were transformed to calculate 30% endpoint titers (ELISA) as described above. For neutralization assays, an infection control was always included. The dilution that would yield 50% reduction of plaques compared with the infection control was estimated by determining the proportionate distance between two dilutions from which an endpoint titer was calculated (Extended Data Figs. 2b, 3b, 4b,c and 6). A statistical comparison between VACV-reactive 30% endpoint titers was performed using a Mann–Whitney *U*-test. A two-tailed *P* value below 0.05 (Figs. 1a and 2d) or 0.0083 (Fig. 1c) after Bonferroni correction for multiple comparisons was considered significant. Correlation of VACV-reactive 30% endpoints titers and MPXV-neutralizing PRNT_50_ titers was evaluated by Spearman’s *r*. Statistical evaluation was done with GraphPad Prism version 9.02.

### Reporting summary

Further information on research design is available in the Nature Research Reporting Summary linked to this article.

## Online content

Any methods, additional references, Nature Research reporting summaries, source data, extended data, supplementary information, acknowledgements, peer review information; details of author contributions and competing interests; and statements of data and code availability are available at 10.1038/s41591-022-02090-w.

### Supplementary information

Reporting Summary

## Data Availability

Data from the present study are not part of public databases but are available upon reasonable request from the corresponding author. Patient-related data not included in the paper may be subject to patient confidentiality and are unavailable due to the analysis of anonymized data. This study used unique materials, which were custom-made for specific analyses (ELISA antigens and virus stocks). Materials are available upon reasonable request, will be released via a material transfer agreement and can otherwise be obtained via the included experimental protocols in the Methods. The MPXV stock is available through the European Virus Archive Global (Ref-SKU: 010V-04721).

## Code availability

No specific codes were written or generated for analysis of the data. Software use has been disclosed.
