# Supplementary material for: Low levels of monkeypox virus-neutralizing antibodies after MVA-BN vaccination in healthy individuals
Source: Nat Med. 2022 Oct 18;29(1):270–8. doi: 10.1038/s41591-022-02090-w (PMC9873555; doi:10.1038/s41591-022-02090-w)
Supplement: Supplementary file 1 — Reporting Summary [file 41591_2022_2090_MOESM1_ESM.pdf]

nature portfolio

Corresponding author(s): Corine H. GeurtsvanKessel
Rory D. de Vries

Last updated by author(s): Oct 12, 2022

Reporting Summary

Nature Portfolio wishes to improve the reproducibility of the work that we publish. This form provides structure for consistency and transparency in reporting. For further information on Nature Portfolio policies, see our Editorial Policies and the Editorial Policy Checklist.

Statistics

For all statistical analyses, confirm that the following items are present in the figure legend, table legend, main text, or Methods section.

n/a	Confirmed	
☐	☒	The exact sample size (*n*) for each experimental group/condition, given as a discrete number and unit of measurement
☒	☐	A statement on whether measurements were taken from distinct samples or whether the same sample was measured repeatedly
☐	☒	The statistical test(s) used AND whether they are one- or two-sided<br>*Only common tests should be described solely by name; describe more complex techniques in the Methods section.*
☒	☐	A description of all covariates tested
☐	☒	A description of any assumptions or corrections, such as tests of normality and adjustment for multiple comparisons
☐	☒	A full description of the statistical parameters including central tendency (e.g. means) or other basic estimates (e.g. regression coefficient) AND variation (e.g. standard deviation) or associated estimates of uncertainty (e.g. confidence intervals)
☐	☒	For null hypothesis testing, the test statistic (e.g. *F*, *t*, *r*) with confidence intervals, effect sizes, degrees of freedom and *P* value noted<br>*Give P values as exact values whenever suitable.*
☒	☐	For Bayesian analysis, information on the choice of priors and Markov chain Monte Carlo settings
☒	☐	For hierarchical and complex designs, identification of the appropriate level for tests and full reporting of outcomes
☐	☒	Estimates of effect sizes (e.g. Cohen's *d*, Pearson's *r*), indicating how they were calculated

Our web collection on statistics for biologists contains articles on many of the points above.

Software and code

Policy information about availability of computer code

Data collection	No software was used, nor were any codes generated.
Data analysis	Statistical evaluation was done with GraphPad Prism v9.02. The number of infected cells in PRNTs was quantified using the Harmony software (version 4.9, Perkin Elmer).

For manuscripts utilizing custom algorithms or software that are central to the research but not yet described in published literature, software must be made available to editors and reviewers. We strongly encourage code deposition in a community repository (e.g. GitHub). See the Nature Portfolio guidelines for submitting code & software for further information.

Data

Policy information about availability of data

All manuscripts must include a data availability statement. This statement should provide the following information, where applicable:

- Accession codes, unique identifiers, or web links for publicly available datasets
- A description of any restrictions on data availability
- For clinical datasets or third party data, please ensure that the statement adheres to our policy

Data from the present study are not part of public databases, but are available upon request to the corresponding author (CHGvK). Patient-related data not included in the paper may be subject to patient confidentiality, and are unavailable due to the analysis of anonymized data. The corresponding author can be contacted via e-mail (c.geurtsvankessel@erasmusmc.nl), responses will be given within a timeframe of 1-2 weeks. Data can be used according to a data use

agreement. Unique materials were used in the study, which were custom-made for specific analyses (ELISA antigens, virus-stocks). Materials are available upon request, will be released via a Material Transfer Agreement and can otherwise be obtained via the included experimental protocols in the Methods section of this manuscript. The MPXV stock is available through EVAg; Ref-SKU: 010V-04721.

Human research participants

Policy information about studies involving human research participants and Sex and Gender in Research.

Reporting on sex and gender

Sex or gender were not considered in study design. Sex was collected in the study design, and equally distributed in the agepanel of sera, and Imvanex- and MVA-H5-vaccinated participants. 97% of the MPX PCR-positive individuals in the PCR-positive panel were male.

Population characteristics

Age, sex and vaccination statuses were the only covariates analyzed in this initial observational study.

(1) agepanel sera: <1974 N=30 male, N=29 female, median age 61 (range 49-85), >1974 N=37 male, N=30 female, median age 30 (range 15-47).

(2) diagnostic sera: <1974 / PCRneg N=14 male, N=5 female, median age 57 (range 51-80); <1974 / PCRpos N=12 male, N=1 female, median age 57 (range 52-65). >1974 / PCRneg N=17 male, N=4 female, median age 29 (range 20-42); >1974 / PCRpos N=19 male, N=0 female, median age 35 (range 21-41).

(3) Imvanex sera: <1974 N=1 male, N=2 female, median age 52 (range 51-62); >1974 N=8 male, N=10 female, median age 30 (range 24-45).

(4) MVA-H5 sera: The original study included both male and female volunteers between 18 and 28 years of age. An exact allocation of sex and age to the selected samples was not possible here.

Recruitment

Sera for the agepanel were randomly selected from the diagnostic serum bank, and pre-selected for the absence of co-morbidities. Sera for the diagnostic panel were selected from individuals that reported potential MPX symptoms, were PCR-tested, and found PCR-positive or PCR-negative. PCR-positive individuals were predominantly male. Sera from both panels were analyzed anonymized, only information on year of birth and sex was collected. Imvanex-vaccinated individuals were part of a biobank protocol. We recruited healthcare workers from the Erasmus MC with a professional risk of being exposed to MPXV, leading to a potential for selection-bias. No samples were specifically selected, all available samples were included in the analyses. MVA-H5 recruitment was previously reported: Kreijtz JH, Goeijenbier M, Moesker FM, et al. Safety and immunogenicity of a modified-vaccinia-virus-Ankara-based influenza A H5N1 vaccine: a randomised, double-blind phase 1/2a clinical trial. Lancet Infect Dis 2014;14:1196-207. We selected sera from N=22 study participants out of an available cohort of N=40 randomly.

Ethics oversight

(1) Age-panel cohort and (2) Diagnostic cohort. The sera in the age-panel were obtained from the serum bank at the Department of Viroscience, Erasmus MC, for validation purposes. The MPX diagnostic serum samples were submitted to Erasmus MC as a diagnostic center for MPX after privacy-coding / pseudonymization by the sender. Both of these cohorts were fully anonymized in agreement with privacy legislation for retrospective studies based on reuse of stored diagnostic samples. (3) Imvanex cohort. Serum samples were obtained from healthcare workers (HCW) who received Imvanex vaccination for safety reasons as employees of a BSL-3 laboratory. Samples were collected on a biobanking study protocol. The Erasmus MC Medical Ethics Committee gave ethical approval for this work performed as part of the COVA study (ethical permit MEC-2014-398). Written informed consent was obtained from all participants. (4) MVA-H5 cohort. The fourth serum panel consisted of samples that were obtained from participants as part of a past clinical phase I vaccination trial with MVA-H5 in two different regimens. The Erasmus MC Medical Ethics Committee gave ethical approval for this work performed in the FluVec-H5 study (ethical permit METC NL37002.000.12, Dutch Trial Registry NTR3401). Written informed consent was obtained from all participants. All relevant permits were submitted with the manuscript.

Note that full information on the approval of the study protocol must also be provided in the manuscript.

Field-specific reporting

Please select the one below that is the best fit for your research. If you are not sure, read the appropriate sections before making your selection.

☒ Life sciences ☐ Behavioural & social sciences ☐ Ecological, evolutionary & environmental sciences

For a reference copy of the document with all sections, see nature.com/documents/nr-reporting-summary-flat.pdf

Life sciences study design

All studies must disclose on these points even when the disclosure is negative.

Sample size

We exclusively report serological experiments performed with patient material. No samples size calculations were performed, the study purely reports observational data on basis of serum availability.

Data exclusions

No data were excluded in the analysis, and all data are available in the (supplemental) figures.

Replication	All samples from each respective experimental panel (age-panel [1], diagnostic panel [2], Imvanex-vaccinated panel [3], and MVA-H5-vaccinated panel [4]) were analyzed simultaneously per assay to counteract batch effects. ELISAs were thoroughly validated via three additional methods: (1) performing ELISAs with additional negative control sera obtained from patients diagnosed with other infectious diseases (Extended Data Figure 1B), (2) side-by-side comparison of results of 'bridging' samples measured in two independent assays (Extended Data Figure 1C), (3) inclusion of the same reference serum on every ELISA plate (Extended Data Figure 1D). All attempts at replication, both for the bridging samples and the reference control sera were successful. This data has bow been included in the manuscript. All neutralization assays were measured in duplicate, and several bridging samples were included as internal controls. All attempts at replication were successful. If samples significantly deviated, also based on the S-curve generated per sample, ELISA or PRNT was repeated.
Randomization	We exclusively report serological experiments performed with patient material. Sera were allocated to a 'group' on basis of year of birth, MPXV PCR, and / or vaccination status.
Blinding	We exclusively report serological experiments performed with patient material. All sera from specific cohorts were measured in a single assay, but during data collection and analysis researchers were blinded to sample information and were only exposed to serum IDs (not revealing group or status information).

Reporting for specific materials, systems and methods

We require information from authors about some types of materials, experimental systems and methods used in many studies. Here, indicate whether each material, system or method listed is relevant to your study. If you are not sure if a list item applies to your research, read the appropriate section before selecting a response.

Materials & experimental systems

n/a	Involved in the study
☐	☒ Antibodies
☐	☒ Eukaryotic cell lines
☒	☐ Palaeontology and archaeology
☒	☐ Animals and other organisms
☐	☒ Clinical data
☒	☐ Dual use research of concern

Methods

n/a	Involved in the study
☒	☐ ChIP-seq
☒	☐ Flow cytometry
☒	☐ MRI-based neuroimaging

Antibodies

Antibodies used	rabbit-anti-VACV-FITC; Abbexa abx023199; polyclonal (1:1000)<br>HRP-conjugated goat-anti-human IgG; Dako / ThermoFisher 31413; polyclonal (1:6000)
Validation	rabbit-anti-VACV-FITC was previously validated in-house (Effects of pre-existing orthopoxvirus-specific immunity on the performance of Modified Vaccinia virus Ankara-based influenza vaccines, Altenburg et al, Sci Rep). Antibody was re-titrated and evaluated side-by-side with other monoclonals and polyclonal human serum before assays reported in the study were performed.

Eukaryotic cell lines

Policy information about cell lines and Sex and Gender in Research

Cell line source(s)	CEF were isolated from 11-day-old chicken embryos and passaged once before use.<br>Baby hamster kidney 21 (BHK-21), HeLa cells, Vero cells, and Calu-3 cells were obtained through ATCC.
Authentication	None of the cell-lines were further authenticated.
Mycoplasma contamination	All cell-lines were tested negative for mycoplasma contamination during regular screening procedures.
Commonly misidentified lines<br>(See ICLAC register)	No commonly misidentified cell lines were used in this study.

Clinical data

Policy information about clinical studies

All manuscripts should comply with the ICMJE guidelines for publication of clinical research and a completed CONSORT checklist must be included with all submissions.

Clinical trial registration	MVA-H5 vaccination trial:<br>Dutch Trial Registry NTR3401 (https://trialsearch.who.int/Trial2.aspx?TrialID=NTR3401)
Study protocol	ethical permit METC NL37002.000.12 from Erasmus MC, not publicly available but the protocol was submitted with the manuscript.
Data collection	Previously reported in: Kreijtz JH, Goeijenbier M, Moesker FM, et al. Safety and immunogenicity of a modified-vaccinia-virus-Ankara-based influenza A H5N1 vaccine: a randomised, double-blind phase 1/2a clinical trial. Lancet Infect Dis 2014;14:1196-207.

Outcomes

Previously reported in: Kreijtz JH, Goeijenbier M, Moesker FM, et al. Safety and immunogenicity of a modified-vaccinia-virus-Ankara-based influenza A H5N1 vaccine: a randomised, double-blind phase 1/2a clinical trial. Lancet Infect Dis 2014;14:1196-207.
